# Matrix Vesicle‐Associated SMS2 as a Novel Mediator of Biomineralization

**DOI:** 10.1002/jex2.70199

**Published:** 2026-11-03

**Authors:** Phillip T. Newton, Mathieu Y. Brunet, Jose G. Marchan‐Alvarez, Outi Mäkitie, Joost C. M. Holthuis

**Affiliations:** ^1^ Department of Women's and Children's Health Karolinska Institutet Stockholm Sweden; ^2^ Astrid Lindgren Children's hospital Stockholm Sweden; ^3^ Folkhälsan Institute of Genetics Folkhälsan Research Center Helsinki Finland; ^4^ Research Program for Clinical and Molecular Metabolism Faculty of Medicine University of Helsinki Helsinki Finland; ^5^ Children's Hospital University of Helsinki and Helsinki University Hospital Helsinki Finland; ^6^ Department of Molecular Medicine and Surgery Karolinska Institutet and Clinical Genetics Karolinska University Laboratory Karolinska University Hospital Stockholm Sweden; ^7^ Molecular Cell Biology Section Department of Biology/Chemistry Osnabrück University Osnabrück Germany; ^8^ Center for Cellular Nanoanalytics Osnabrück University Osnabrück Germany

**Keywords:** growth plate, matrix vesicle, mineralization, SGMS2, SMS2, sphingomyelin

## Abstract

*SGMS2*, a gene encoding plasma membrane‐resident sphingomyelin synthase 2, SMS2, has emerged as an important determinant of skeletal ossification. Heterozygous *SGMS2* variants are associated with a broad spectrum of bone phenotypes, ranging from rickets‐like deformities in childhood to early‐onset osteoporosis, implicating SMS2 in mineralization while the underlying mechanisms are incompletely resolved. As SMS2 activity alters the balance of membrane lipids such as ceramide and sphingomyelin, the effects of SMS2 dysfunction on the skeleton are likely to be multifactorial and distributed across multiple cell types and subcellular compartments. Our recent work has identified SMS2 as a key component of growth plate‐derived matrix vesicles, nanoscale membrane‐bound particles released by skeletal cells that nucleate mineral deposition. Consistent with a functional role in this compartment, inhibition of SMS2 reduced the mineralization capacity of isolated matrix vesicles *ex vivo*. Collectively, these observations suggest that SMS2 contributes directly to biomineralization within matrix vesicles. Here, we place this emerging finding in the broader context of skeletal biology, disease mechanism, and therapeutic potential.

## 1 Introduction

Extracellular vesicles (EVs) are membrane‐bound, fluid‐filled sacs in the nanometre size range that are thought to be released by all cells (Wiklander et al. 2019). Biomineral nucleation was one of the earliest functional roles ascribed to any EV, occurring in the lumen of a specialized class of EVs released by chondrocytes, osteoblasts, and other mineralizing cells, called matrix vesicles (MVs) (Bottini et al. 2018; Cui et al. 2016). The pioneering work of Bonucci, Clarke Anderson and Ali in the middle of the last century identified these membrane‐delimited nanoparticles as the likely originators of mineralization based on exquisite electron microscopy images (Anderson 1969; Anderson et al. 1970; Bonucci 1967). Subsequent work has revealed they range in size from 40 ‐ 1000 nm in diameter (Bottini et al. 2018) and contain enzymes that enable mineralization. The actions of these enzymes in both the degradation of the mineralization inhibitor, pyrophosphate (PP_i_), and the accumulation of inorganic phosphate (P_i_), are crucial for a permissive P_i_/PP_i_ ratio to enable biomineralization. The build‐up of both P_i_ and calcium ions within the lumens of MVs facilitates the earliest steps of amorphous calcium/phosphate precipitates, a precursor to hydroxyapatite crystallization (Wuthier 2011).

Calcium ion availability for mineralization is controlled by annexins and L‐type Ca^2+^ channels (Bottini et al. 2018; Wuthier 2011), whereas phosphate, generated in the extravesicular space by the actions of tissue non‐specific alkaline phosphatase (ALPL) (Anderson et al. 1970; Majeska and Wuthier 1975) and/or ectonucleotide pyrophosphatase/phosphodiesterase‐1 (ENPP1) (Ciancaglini et al. 2010), is pumped into the MV lumen by Phosphate transporter‐1 (encoded by SLC20A1), a sodium‐dependent phosphate cotransporter (Figure 1) (Cominal et al. 2010; Jono et al. 2000). However, an additional source of phosphate ions is generated directly within the MV lumen by the enzyme PHOSPHO1 (Ciancaglini et al. 2010; MacRae et al. 2010; Roberts et al. 2007), which cleaves the phosphate ion from phosphoethanolamine and phosphocholine. Upstream of this, phosphocholine itself is produced by the enzyme SMPD3 (Stoffel et al. 2007), a phosphodiesterase that hydrolyses sphingomyelin (SM) to generate ceramide in the inner leaflet of MVs (Bottini et al. 2018; Stoffel et al. 2007). Phosphocholine is also generated by choline kinase beta, encoded by CHKB (Li et al. 2014), and by ENPP6 (Dillon et al. 2021) during endochondral ossification. Extensive in vivo evidence indicates that impaired expression or function of these genes leads to skeletal under‐mineralization phenotypes in humans and animal models (Aubin et al. 2005; Bourne et al. 2026; Dillon et al. 2021; Huesa et al. 2011; Stoffel et al. 2007; Wuthier 2011; Yadav et al. 2016). While mice harboring a global *Chkb* knockout exhibit a skeletal phenotype, mineralization is only mildly affected (Li et al. 2014), suggesting the presence of functional redundancy and/or compensatory mechanisms, a phenomenon that has been widely reported across multiple components of the mineralization machinery (Bottini et al. 2018; Bourne et al. 2026; Ciancaglini et al. 2010; Yadav et al. 2016).

**FIGURE 1:**
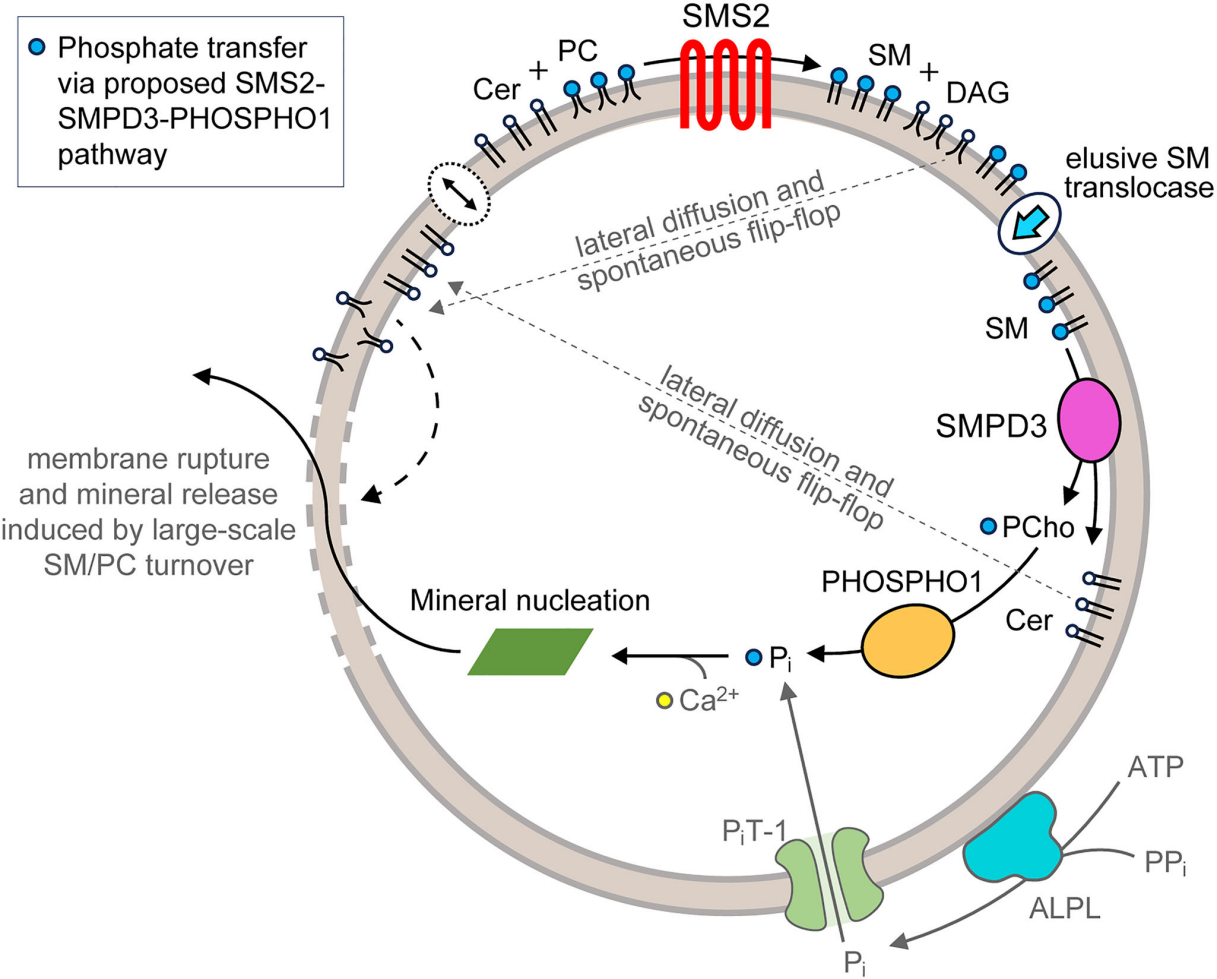
Schematic positioning the crucial role of SMS2 in phosphate ion availability within matrix vesicles. While SMS2 is actively involved in mineralization within matrix vesicles, and its enzymatic product, sphingomyelin, can be used as a substrate by SMPD3 to eventually generate phosphate ions, this would require flip‐flop from the outer to the inner membrane leaflet. The continuous shuttling of phosphate from the membrane lipids to enable amorphous calcium phosphate crystal formation, and eventual hydroxyapatite growth, can contribute to membrane rupture and mineral propagation within the extracellular matrix. Abbreviations: ATP, adenosine triphosphate; Cer, ceramide; DAG, diacylglycerol; ENPP1, ecto‐nucleotide pyrophosphatase/phosphodiesterase 1; PC, phosphatidylcholine; PCho, phosphocholine; P_i_, inorganic phosphate; PiT‐1, phosphate transporter 1; PP_i_, pyrophosphate; SM, sphingomyelin; SMS2, sphingomyelin synthase 2; SMPD3, Sphingomyelin phosphodiesterase 3. The figure was modified from (Bottini et al. 2018).

### 1.1 SMS2 Links Sphingolipid Metabolism to Membrane Organization and Signaling

Sphingomyelin synthase 2 (SMS2) is an enzyme also known as phosphatidylcholine:ceramide cholinephosphotransferase 2, which is encoded by the *SGMS2* gene (Huitema et al. 2004; UniProt Consortium 2026). Human genetic analysis has identified impaired skeletal mineralization as a common feature of *SGMS2*‐associated diseases (Pekkinen et al. 2019). Heterozygous pathogenic variants in *SGMS2* give rise to a broad clinical spectrum of skeletal disorders, ranging from childhood‐onset osteoporosis with low bone mineral density to severe spondylometaphyseal dysplasia with neonatal fractures, long‐bone deformities, short stature, and characteristic calvarial “doughnut‐shape sclerotic” lesions. In transiliac biopsies from adults with *SGMS2*‐related osteoporosis, bone histomorphometry and quantitative backscattered electron imaging showed low and heterogeneous matrix mineralization, prolonged mineralization lag time, and profound disorganization of the osteocyte lacuno‐canalicular network, including enlarged lacunae and sparse, disordered canaliculi (Mäkitie et al. 2021). Although less thoroughly explored, *SGMS2*‐related spondylometaphyseal dysplasia presents, in addition to skeletal fragility, also rickets‐like features, including widened growth plates, severe growth failure and progressive skeletal curvature (Pekkinen et al. 2019). Notably, the skeletal consequences of pathogenic *SGMS2* variants may not be limited to under‐mineralization. A recently reported family carrying a novel heterozygous *SGMS2* frameshift variant exhibited diffuse high bone mass together with recurrent fragility fractures (Patra et al. 2025). Taken together, the available clinical, genetic, and histological data position *SGMS2* as an important regulator of mineral deposition with broad consequences on the skeleton. What has remained unclear, however, is where SMS2 acts within skeletal cells and matrix to exert these effects on biomineralization.

SMS2 predominantly resides at the plasma membrane. The enzyme contains six membrane‐spanning helices, and when positioned on the cell surface, its active site faces the extracellular side of the membrane (Kuo and Hla 2024; Wang et al. 2024). The enzymatic function of SMS2 is to transfer the phosphocholine headgroup from phosphatidylcholine to ceramide, thereby generating sphingomyelin (SM) and diacylglycerol (DAG) (Taniguchi and Okazaki 2020; Ternes et al. 2009), although it can also catalyse the formation of ceramide phosphoethanolamine (CPE) (Ternes et al. 2009). Due to these functions, SMS2 activity can influence membrane properties and cell behaviour, balancing the local levels of ceramide and phosphatidylcholine with SM and DAG. Interestingly, the generation of DAG as a by‐product of SM production may link SMS2's bioactivity to further downstream signalling leading to cellular responses in bone cells. For example, the use of DAG has demonstrated bone health benefits in a mouse model of obesity‐induced osteoporosis by modulating key gene expression of bone formation and resorption (Yue et al. 2026), thus, suggesting the possibility of supporting secondary cues linked to the bioactivity of SMS2.

While the literature has predominantly highlighted the proteome of MVs, growing evidence points to membrane lipid composition and turnover as critical factors in their formation and function. Originally, just a few years after their discovery, MV preparations were shown to be enriched in specific lipid species including SM, cholesterol and phosphatidylserine (Peress et al. 1974). This suggested a shared lipid signature between MVs and lipid rafts, specialized membrane microdomains that were first described in 1997 by Simons and Ikonen (Simons and Ikonen 1997). As GPI‐anchored ALPL and Annexin A6 are key MV proteins that are associated with lipid rafts, there appears to be a direct connection between SM and protein compositions of MVs. As such, Hayann et al. studied osteoblast‐derived MVs (MC3T3‐E1 model) and the influence of GW4869, a sphingomyelin phosphodiesterase/neutral sphingomyelinase inhibitor. Interestingly, the researchers reported that MVs secreted by GW4869‐treated osteoblasts contained significantly lower levels of SM and ceramide as well as reduced ALPL recruitment to MVs (Hayann et al. 2026), thus, highlighting an important interplay between sphingolipids and MV function.

As sphingomyelin and ceramide significantly influence membrane fluidity and curvature (Lima et al. 2025), lipid raft formation (Bieberich 2018; Pike 2003), cholesterol distribution (Kuo and Hla 2024), vesicle budding (Trajkovic et al. 1979) and cargo sorting (Shah et al. 2026), it seems likely that changes in SMS2 activity would have immediate consequences for membrane architecture, organisation and signalling.

### 1.2 SMS2 Has a Functional Role in Biomineralization From Within Matrix Vesicles

In our recent work, we applied spatially resolved transcriptomics to human growth plate biopsies from healthy individuals with idiopathic tall stature, which revealed that *SGMS2* is expressed by hypertrophic zone (HZ) chondrocytes (Avijgan et al. 2026). This finding was corroborated by scRNAseq analysis of human growth plate chondrocytes, where *SGMS2* expression was identified at a similar point in the differentiation trajectory as the HZ markers, *ALPL* and *COL10A1* (Chu et al. 2026). Given the growth plate abnormalities present in individuals carrying *SGMS2* variants, and that mineralization occurs within the HZ, we prioritized *SGMS2* for deeper investigation.

Considering that (i) the lipid composition of MVs deriving from growth plate has been estimated as 41.8 ± 2.5 % phosphatidylcholine (Wuthier 2011), and (ii) SMPD3 utilizes sphingomyelin as a substrate within MVs, we hypothesized that SMS2 could be directly involved in mineralization within MVs. To test this hypothesis, we isolated murine growth plate‐derived MVs, which contained relatively abundant SMS2 when analysed by liquid chromatography electrospray tandem mass spectrometry. Using an established turbidity‐based MV‐mineralization assay (Cominal et al. 2010; Wu et al. 1997), pharmacological inhibition of the enzymatic activity of SMS2 delayed mineral formation, supporting a functional role for SMS2 in promoting MV‐initiated mineralization (Avijgan et al. 2026).

### 1.3 How Could SMS2 Participate in Mineralization Within Matrix Vesicles?

The Wuthier lab previously isolated chick growth plate‐derived MVs to monitor temporal changes in phospholipid species during *ex vivo* mineralization (Wu et al. 2002). This revealed that total phospholipids were gradually converted to non‐phospholipids as mineralization progressed, suggesting that phosphate groups are released from membrane lipids and shuttled into the vesicle lumen to be utilised for mineralization. More specifically, the authors observed a gradual depletion of both phosphatidylcholine and sphingomyelin as mineralization progressed, providing a broader perspective to how the function of SMS2 may fit with overall phosphate shuttling into eventual mineral (Figure 1).

While phosphocholine released by SMPD3 provides a direct source of phosphate to promote bone mineralization, SMS2 catalyses a reaction whereby phosphocholine is cleaved from phosphatidylcholine and subsequently transferred onto ceramide to generate sphingomyelin and diacylglycerol. Even though SMS2 can also catalyse the reverse reaction (Huitema et al. 2004), the enzyme is unable to release phosphocholine and instead forms a histidine‐phosphocholine intermediate during the reaction cycle. However, by regenerating sphingomyelin from ceramide released by SMPD3 using phosphatidylcholine as head group donor, SMS2 may serve to replenish the sphingomyelin pool used by SMPD3. This coupling of SMPD3 and SMS2 enzymatic activities would enable a continuous supply of lipid‐derived phosphate required for normal bone mineralization. Furthermore, large‐scale metabolic conversion of sphingomyelin and phosphatidylcholine into ceramide and diacylglycerol would reduce membrane stability and facilitate vesicle lysis, a process necessary for mineral propagation throughout the extracellular matrix (Figure 1).

While it seems plausible that SMS2‐derived sphingomyelin could be used as a phosphate carrier to be used for mineralization, the enzymatic activity of SMS2 generates sphingomyelin in the outer leaflet of the MV membrane, whereas SMPD3 is thought to act on the inner leaflet of the membrane (Bottini et al. 2018; Kobayashi and Menon 2018). Hence, sphingomyelin would need to flip‐flop. For lipids with a bulky polar head group, flip‐flop occurs passively at very low rates and requires the assistance of a scramblase or flippase. In our own dataset, we detected the scramblase Plscr1 (Avijgan et al. 2026; Damek‐Poprawa et al. 2006). However, whether Plscr1 participates in the flip‐flop of sphingomyelin during bone mineralization remains to be established. While it remains unclear exactly how sphingomyelin is transported from the outer to the inner leaflet of the plasma membrane (Kobayashi and Menon 2018), PMP2/FABP8 can facilitate this process in a PI(4,5)P2‐dependent manner (Abe et al. 2021). Interestingly, lysosomal damage triggers a rapid Ca^2+^‐dependent sphingomyelin scrambling, potentially involving a TMEM16F‐related scramblase (Niekamp et al. 2022). TMEM16F serves as the primary Ca^2+^‐activated phospholipid scramblase in the plasma membrane. Although inactivation of TMEM16F leads to decreased mineral deposition in skeletal tissues (Ehlen et al. 2013), a recent study has cast doubt on the assumption that TMEM16F is responsible for catalysing sphingomyelin flip‐flop during bone mineralization (Le et al. 2026). On the other hand, sphingomyelin flip‐flop may not be necessary for its phosphocholine head group to contribute to mineralization; the ectoenzyme, ENPP6, generates phosphocholine in the extracellular milieu through hydrolysis of lysophospholipids (Dillon et al. 2021). Since ENPP6‐deficient mice experience skeletal under‐mineralization, and phosphocholine was recently found to be a substrate for ALPL (Drevet Mulard et al. 2025), extravesicular phosphocholine appears to be an important source of P_i_ for biomineralization. However, while it is also conceivable that extracellular phosphate could be derived from sphingomyelin, we did not identify known soluble sphingomyelinases (i.e. Smpd1, Enpp7) in the isolated MV preparations, in our study (Avijgan et al. 2026).

In contrast to the slow spontaneous movement of phospholipids between membrane leaflets, lateral diffusion within the leaflet is rapid. This raises the possibility that SMS2‐mediated sphingomyelin production could also regulate mineralization independently of phosphate ion metabolism. Such a mechanism is plausible, as sphingomyelin and its metabolites play central structural and functional roles in membranes, as described (Bieberich 2018; Kuo and Hla 2024; Lima et al. 2025; Pike 2003; Trajkovic et al. 1979). In this context, one plausible model is that SMS2‐dependent sphingomyelin microdomains help position or prolong the stability of mineralization machinery—including proteins such as ENPP1, ALPL, and annexins—as the membrane is being catabolized (Damek‐Poprawa et al. 2006; Hayann et al. 2026; Wu et al. 2002).

The central role of lipids in MV‐mediate mineralisation has also been reported by studies on MV mimetics aiming to replicate features of MVs using bottom‐up approaches. As such the development of proteoliposomes has allowed the study of simpler models of known and homogenous features compared to their naturally derived counterparts (Bolean et al. 2015; Veschi et al. 2022). A critical aspect of these studies focuses on the lipid composition of the synthetic MV as it is controlled parameter. Simão and colleagues generated ALPL‐harboring liposomes while varying their lipid composition (ratios of PC/SM/Cholesterol) to evaluate their ability to nucleate minerals via turbidity assay. Notably, they reported that ALPL‐MV mimetic nucleated more minerals in the following order PC > PC:SM (9:1) > PC:Chol:SM (8:1:1) > PC:Chol (9:1), suggesting that the presence of SM alone could reduce their ability to produce minerals (Simão et al. 2019). These data may also suggest that the modulation of the lipid composition alone is not a sufficient condition to improve the pro‐mineralizing capacity of MVs and may require a more complex model involving the SMS2‐SMPD3‐PHOSPHO1 axis to further utilize the presence of sphingolipids.

### 1.4 Implications of Matrix Vesicle–Associated SMS2 in Biomineralization

Since the MV experiments discussed here are based on murine material (Avijgan et al. 2026), it is interesting to note the phenotype of global *Sgms2* knockout mice. Importantly, murine genetics show that *Sgms1* rather than *Sgms2* is indispensable for osteoblast differentiation and bone formation (Matsumoto et al. 2019), whereas *Sgms2* deficiency alone does not recapitulate the severe *SGMS2* skeletal phenotypes observed in humans (Matsumoto et al. 2019; Pekkinen et al. 2019). Whether or not the mild changes in alizarin red stained skeletal preparations reflect a *bona fide SGMS2*‐associated phenotype (Matsumoto et al. 2019), it is clear that the consequences of loss of *Sgms2* function in mice is less severe than those reported for pathogenic *SGMS2* variants in humans. Interestingly, *SGMS2* variants linked to the most severe bone phenotypes, p.Ile62Ser and p.Met64Arg, give rise to SMS2 enzymes that retain full catalytic activity but are defective in ER export (Pekkinen et al. 2019). Cells harboring these variants accumulate sphingomyelin in the ER and display a disrupted transbilayer sphingomyelin asymmetry (Sokoya et al. 2022). These aberrant sphingomyelin distributions also occur in patient‐derived fibroblasts and are accompanied by imbalances in cholesterol organization, glycerophospholipid profiles, and lipid order in the secretory pathway. How these imbalances in membrane lipid organization affect bone mineralization remains to be established.

For mineralization to spread across the extracellular matrix, growing crystals need to penetrate/rupture the MV membrane; however, the underlying mechanisms are uncertain (Hasegawa et al. 2017). As previously postulated (Wu et al. 2002), degrading the membrane's phospholipids will alter its physical properties, such as curvature and lipid packing, potentially facilitating vesicle lysis. In this context, large scale turnover of both PC and SM involving SMS2 could participate in the global de‐phosphorylation of the MV membrane, and its eventual membrane rupture and mineral propagation.

Whether or not our findings can be extrapolated to all mineralizing MVs warrants further investigation. The MVs we isolated were among small EVs (≤ 200 nm in diameter), representing only a subset of all MVs, and the presence of SMS2 in other mineralizing MV populations is yet to be determined. Considering that *SGMS2* is a highly evolutionarily conserved gene whose appearance coincides with the that of Euteleostomi (bony vertebrates) (Wang et al. 2024), general scope of SMS2 involvement in MV‐associated mineralization merits further experimental studies. If SMS2 proves to be a universal marker and functional component of mineralizing MVs, modulation of its activity could represent a novel therapeutic strategy for disorders of impaired or excessive mineralization, such as osteoporosis and osteopetrosis. Such an approach is particularly attractive because MV‐associated SMS2 would be accessible extracellularly, potentially avoiding the need for intracellular drug delivery and minimizing interference with essential cellular membrane biogenesis pathways. However, SMS2 may not be specific to mineralizing MVs as SMS2 has been detected in other extracellular vesicle populations, including exosomes derived from mesenchymal stem cells (Xu et al. 2023). These observations raise the possibility that SMS2 may have broader roles in EV biology beyond mineralization, highlighting the need for future studies to define both its specificity and functional diversity across distinct EV populations.

## 2 Conclusion

We propose that SMS2, SMPD3, and PHOSPHO1 together define a pathway in which sphingomyelin metabolism is functionally coupled to phosphate shuttling from MV membrane phospholipids to hydroxyapatite during MV‐mediated mineralization. This framework offers new perspectives into phenotypes caused by *SGMS2* variants and the physiological mechanisms of vertebrate mineralization.

## Author Contributions

**Phillip T. Newton**: conceptualization, investigation, funding acquisition, supervision, project administration, visualization, writing – original draft, writing – review and editing. **Mathieu Y. Brunet**: visualization, writing – original draft. **Jose G. Marchan‐alvarez**: writing – original draft. **Outi Mäkitie**: writing – review and editing. **Joost C. M. Holthuis**: visualization, writing – review and editing.

## Conflicts of Interest

The authors declare no conflicts of interest.

## Generative AI Statement

During the preparation of this work the author(s) used Microsoft CoPilot within Karolinska Institutet´s data protection environment in order to screen literature and edit language. After using this tool/service, the authors reviewed and edited the content as needed and take full responsibility for the content of the published article.
